# Childbirth experience in induced labor: A prospective study using a validated childbirth experience questionnaire (CEQ) with a focus on the first birth

**DOI:** 10.1371/journal.pone.0274949

**Published:** 2022-10-06

**Authors:** Katariina Place, Leena Rahkonen, Niina Verho-Reischl, Katti Adler, Seppo Heinonen, Heidi Kruit

**Affiliations:** Department of Obstetrics and Gynecology, University of Helsinki and Helsinki University Hospital, Helsinki, Finland; University of the Witwatersrand, SOUTH AFRICA

## Abstract

**Objective:**

First birth and labor induction are risk factors for negative childbirth experiences. As labor inductions are increasing, research into this high-risk group’s childbirth experiences is important. We aimed to investigate whether nulliparity or factors related to labor induction, labor, and delivery explain the association.

**Methods:**

This was a prospective study of 711 women undergoing labor induction at Helsinki University Hospital, Finland, between January 1, 2019, and January 31, 2020. The participants answered the Childbirth Experience Questionnaire (CEQ) after delivery (response rate 69.4%). The patient characteristics and delivery outcomes were collected from patient records. We analyzed the results for nulliparous and parous women.

**Results:**

The mean CEQ scores were 2.9 (SD 0.5) for nulliparous women (n = 408) and 3.2 (SD 0.5) for parous women (n = 303), on a scale of 1–4; higher scores represent more positive experiences. However, 7.3% of the women had negative childbirth experiences (8.8% nulliparous; 5.3% parous, p = 0.08). Negative experiences were associated with a cesarean section (OR 6.7, 95% CI 1.8–9.3, p < 0.001) and a hemorrhage ≥ 1500 ml in vaginal delivery (OR 2.8, 95% CI 1.1–7.5, p = 0.03). In the separate CEQ domains analyses, nulliparity was associated with negative experiences in the “Own Capacity” domain (OR 1.6, 95% CI 1.0–2.4, p = 0.03). Cervical ripening, oxytocin use, and daytime delivery were associated with negative experiences in at least one domain, whereas epidural or spinal analgesia was regarded positively in two domains and negatively in one.

**Conclusions:**

Nulliparous women undergoing labor induction risk negative childbirth experiences mainly due to labor and delivery-related factors, similar to parous women. Their perceptions of their capacity and preparedness for labor and delivery should be enhanced antenatally. An effective labor induction protocol promoting as high a rate of vaginal delivery as possible and preparedness to promptly respond to postpartum hemorrhage are key for avoiding negative childbirth experiences.

## Introduction

The childbirth experience profoundly affects the mother´s health and [1, 2]. In Western societies where maternal and neonatal mortality and morbidity rates are low, maternal childbirth experience has become a significant factor in maternity care [3]. Most women want a positive childbirth experience in which safety and psychosocial well-being are equally valued [3]. A negative childbirth experience has been associated with delaying or preventing women from having more children [4], and as several European countries have low birth rates below the replacement rate of 2.1 [5], positive childbirth experiences for women are in the interest of both the individual and society. For example, Finland’s population structure is among the oldest in Europe [6].

Labor induction is a known pre-labor risk factor for a poor childbirth experience, and in Finland, 34.3% of all labors were induced in 2020 [7–10]. Another known pre-labor risk factor is nulliparity, and in Finland, 2020, first births comprised 42.5% of all births [7, 9–11]. Other acknowledged labor-related risk factors for poor childbirth experience are operative delivery (cesarean section, vacuum extraction, or forceps), a long labor (≥12 hours), oxytocin use, maternal complications, hemorrhage, and neonatal complications such as admission to an intensive care unit [7–9, 11–15]. Effect of time of delivery is uncertain, but daytime delivery is often seen as beneficial [16, 17].

As women giving birth may be at risk for a negative childbirth experience even before labor, and some encounter additional hardships along the way, research on how the childbirth experience is formed in these risk groups is necessary.

We aimed to investigate whether nulliparity itself or factors related to labor induction and labor and delivery outcomes explain the increased risk of a negative childbirth experience in nulliparous women. We also aimed to discover factors in our maternity care processes that could be improved to enhance the woman-centeredness and user-perceived value of healthcare.

## Materials and methods

This prospective study on women undergoing labor induction was conducted at the Department of Obstetrics and Gynecology, Helsinki University Hospital, between January 1, 2019, and January 31, 2020. The study protocol was approved by the institutional review board of the hospital region (Helsinki and Uusimaa Hospital District Committee for Obstetrics and Gynecology) (HUS/3172/2018). All the participating women gave their written informed consent after receiving written and oral information about the study.

In Finland, all women give birth at public hospitals, and antenatal care is free or low-cost for everyone. Nulliparous women are also offered childbirth coaching, which is intended to provide basic knowledge about childbirth. In addition, some women opt to take supplementary private paid classes on various aspects of pregnancy and childbirth.

**Fig 1:**
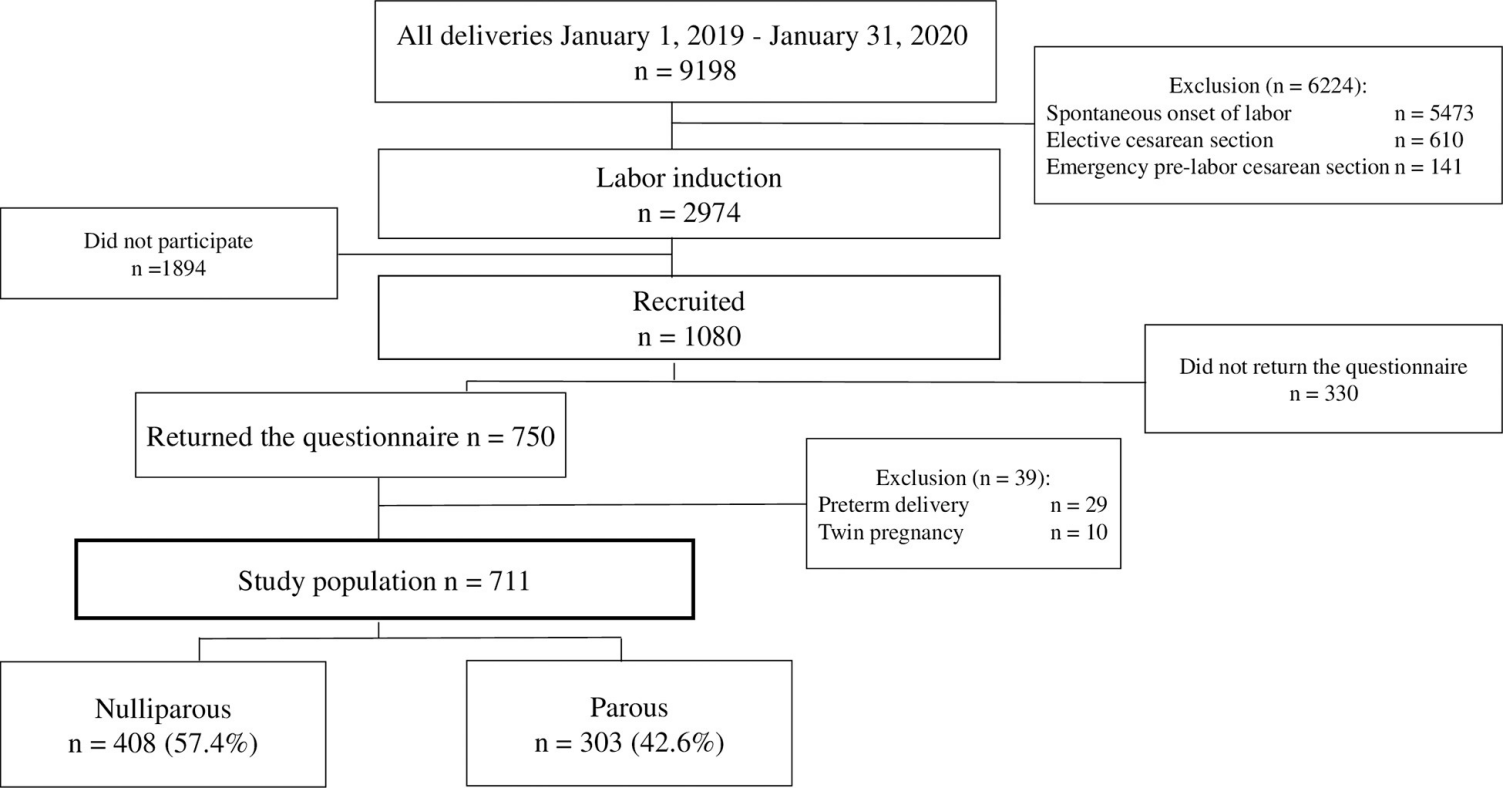
Study population.

We included nulliparous and parous women with viable singleton pregnancies undergoing labor induction who had sufficient written and oral understanding of Finnish, Swedish, or English. The exclusion criteria were multiple gestation and prematurity (< 34 gestational weeks). All women with no contraindication for vaginal delivery and scheduled for labor induction received oral and written study information upon admission to the labor induction unit and were asked to participate. Fig 1 presents the study design and population.

We used the multidimensional Childbirth Experience Questionnaire (CEQ) to evaluate childbirth experience [13, 14]. The CEQ was originally created and validated in Sweden, which has a comparable cultural environment and similar obstetric practices to those in Finland. The questionnaire was handed to women upon admission to the labor induction unit after they were informed about the study and provided their written informed consent. The questionnaire was available in Finnish, Swedish, and English and the women were asked to return it within one month post childbirth by mail or email. The questionnaire has been validated in all the languages used in this study [8, 13, 14].

In the CEQ, items are grouped into four domains: “Own Capacity,” “Professional Support,” “Perceived Safety,” and “Participation.” Of the 22 items, 19 are scored on a four-point Likert scale (totally agree = 4, mostly agree = 3, mostly disagree = 2, and totally disagree = 1). Three items are assessed with a visual analog scale (VAS, items 20–22), and the VAS scale scores are changed to categorical values: 0–40 = 1, 41–60 = 2, 61–80 = 3, and 81–100 = 4. Negatively worded statements and the item on labor pain are reversed (items 3, 5, 8, 9, and 20) so that higher scores reflect a more positive experience, as for the other items. The domain scores are computed as the means of individual scores within the domain, and the total CEQ score is computed similarly [13, 14].

The primary outcome of the study was the childbirth experience measured by the CEQ in women whose labor was induced. Nulliparous and parous women were analyzed as separate cohorts. The secondary outcomes were factors affecting a negative childbirth experience in individual CEQ domains (the most negative quartile of the scores given) and as a whole (all CEQ domain scores in the most negative quartile of the scores given). These analyses were performed in a case-control setting. We had no sample size calculations prior to commencing the study, as we planned this to be a one-year cohort study.

To assess the usability of the CEQ in our study population, we examined the questionnaire´s floor and ceiling effects (i.e., the percentages of the most negative and the most positive responses given). Percentages higher than 15 indicate unsuitability within the population studied, because if too many respondents choose the extreme response, no distinction between the respondents is possible [18].

The patient characteristics and delivery outcomes were collected from patient records and linked anonymously by identification number to the results of the CEQ. The indications for labor induction included post-term pregnancy, prolonged pre-labor rupture of membranes, diabetes, hypertension or preeclampsia, maternal reasons (fear of childbirth, maternal request, maternal medical condition unrelated to pregnancy, and maternal substance abuse) and fetal reasons (complications in an earlier pregnancy and delivery, intrauterine growth restriction, cholestasis of pregnancy, breech presentation, macrosomia, oligohydramnios, polyhydramnios, fetal heart defect or other anomaly, successful external cephalic version for an unstable lie, Rhesus immunization, decreased fetal movements, and other unspecified risks in pregnancy). Maternal complications in labor included uterine rupture, manual removal of a retained placenta, sphincter injury, shoulder dystocia, and intrapartum infection (defined as maternal fever ≥ 38°C during labor and at least one of the following: fetal or maternal tachycardia, uterine tenderness, purulent discharge, or white cell count > 15 × 10^9^/l). The interval between the start of labor induction and birth defined the induction to delivery interval. Regular contractions and cervical dilations of ≥ 6 cm defined onset of the active phase of labor [19]. Failed labor induction was diagnosed if the labor did not progress despite ruptured membranes and 12–18 hours of oxytocin administration [20]. Labor arrest was diagnosed in the first stage of labor if there were adequate contractions for ≥ 4 hours, cervical dilation of ≥ 6 cm, and ruptured membranes, but no progress [21]. Labor arrest was diagnosed in the second stage of labor if delivery failed at full cervical dilation and ≥ 1 hour of active pushing or if failed operative vaginal delivery failed [21]. Time of day during delivery was divided into three categories: night 00:00–07:59, day 08:00–15:59, and evening 16:00–23:59.

Statistical analyses were performed using IBM SPSS Statistics for Windows, version 27.0. Categorical variables were compared using the Chi-square test and Fisher’s exact test when appropriate. Continuous variables were analyzed by a t-test when the data followed normal distribution and by the Mann-Whitney U test when it did not. Logistic regression analyses were performed to assess the odds ratios for a negative childbirth experience [22]. In these multivariable analyses, adjusted odds ratios (OR) with 95% confidence intervals (CI) were calculated by modeling the data to control for possible confounding factors. These variables, first used in the multivariable analyses for a negative childbirth experience in each individual CEQ domain, were chosen by assessing the previous literature for known risk factors for a negative childbirth experience and using stepwise logistic regression analyses (all factors are presented in Tables 1 and 2) to find additional risk factors. All variables statistically significantly related to a negative experience in at least one domain were chosen for analysis of a negative childbirth experience in the whole CEQ. All variables used in the final multivariable analyses are shown in tables with univariable analyses (Tables 5 and 6). A p-value < 0.05 was considered statistically significant. Missing data, if any, were handled by pairwise exclusion when possible (Tables 1–4) and in logistic regression by listwise exclusion (Tables 5 and 6).

**Table 1:** Characteristics of the study population (N = 711).

	Nulliparous		Parous		
	n = 408	57.4%	n = 303	42.6%	p-value
Maternal age ≥ 37 years	90	22.1	110	36.3	< 0.001
Maternal height < 164 cm	119 ^a^	29.2	91	30.0	0.85
Body mass index ≥ 30 kg/m^2^	82 ^b^	20.1	72	23.8	0.25
In vitro fertilization	38	9.3	21	6.9	0.26
Smoking	23	5.6	18	5.9	0.87
Pregestational diabetes (types I and II)	16	3.9	12	4.0	0.98
Gestational diabetes	108	26.5	106	35.0	0.01
Breech presentation	9	2.2	3	1.0	0.25
Late-term pregnancy (≥ 41 weeks)	182	44.6	96	31.7	< 0.001
Bishop score at start of labor induction < 3	203	49.8	124	40.9	0.02
Cervical ripening	366 ^c^	89.7	253 ^d^	83.5	0.02
Indication for labor induction					
Post-term pregnancy	153	37.5	73	24.1	< 0.001
Prolonged pre-labor rupture of membranes	103	25.2	44	14.5	< 0.001
Diabetes	45	11.0	68	22.4	< 0.001
Preeclampsia	38	9.3	17	5.6	0.07
Maternal reason	10 ^e^	2.5	39 ^f^	12.9	< 0.001
Fetal reason	59 ^g^	14.5	62 ^h^	20.5	0.04

a missing values n = 3

b missing values n = 1

c balloon catheter n = 244, oral misoprostol n = 113, vaginal misoprostol n = 5, balloon catheter with oral misoprostol n = 3, vaginal dinoprostone n = 1

d balloon catheter n = 218, oral misoprostol n = 33, vaginal misoprostol n = 1, balloon catheter with oral misoprostol n = 1

e fear of childbirth n = 6, maternal request n = 2, maternal medical condition unrelated to pregnancy n = 1, maternal substance abuse n = 1

f fear of childbirth n = 24, maternal request n = 10, maternal medical condition unrelated to pregnancy n = 4, maternal substance abuse n = 1

g intrauterine growth restriction n = 13, cholestasis in pregnancy n = 13, breech presentation n = 8, macrosomia n = 7, oligohydramnios n = 7, fetal heart defect or other anomaly n = 5

successful external cephalic version for an unstable lie n = 2, Rhesus immunization n = 2, decreased fetal movements n = 1, other unspecified risks in pregnancy n = 1

h complications in an earlier pregnancy and delivery n = 13, intrauterine growth restriction n = 12, cholestasis in pregnancy n = 6, macrosomia n = 7, oligohydramnios n = 3

fetal heart defect or other anomaly n = 9, successful external cephalic version for an unstable lie n = 4, Rhesus immunization n = 4, other unpecified risks in pregnancy n = 3, polyhydramnios n = 1

**Table 2:** Delivery and neonatal outcomes (N = 711).

	Nulliparous		Parous		
	n = 408	57.4%	n = 303	42.6%	p-value
Oxytocin use in labor induction or augmentation	369	90.4	205	67.7	< 0.001
Epidural or spinal analgesia ^a^	358	88.0	215	71.0	< 0.001
Vaginal delivery	205	50.2	257	84.8	< 0.001
Operative vaginal delivery by vacuum extraction	72	17.6	15	5.0	< 0.001
Cesarean section	131	32.1	31	10.2	< 0.001
Fetal distress	42	32.1	9	29.0	0.74
Failed induction	43	32.8	14	45.2	0.20
Labor arrest	39	29.8	5	16.1	0.13
Other	7^b^	5.3	3^c^	9.7	0.41
Episiotomy	97	24.4	24	8.3	< 0.001
Maternal complication in labor					
Uterine rupture	0	0	1	0.3	
Manual removal of retained placenta	7	1.7	11	3.6	0.11
Sphincter injury	10	2.5	3	1.0	0.15
Shoulder dystocia	3	0.7	4	1.3	0.44
Intrapartum infection	30	7.4	8	2.6	0.006
Postpartum infection	13^d^	3.2	4^e^	1.3	0.11
Hemorrhage ≥ 1500 ml in vaginal delivery	29	7.1	22	7.3	0.94
Induction to delivery interval ≥ 24 h	264	64.7	91	30.0	< 0.001
Duration of labor ≥ 12 h	168	41.2	24	7.9	< 0.001
Delivery during the night (00:00–07:59)	152	37.3	92	30.4	0.06
Delivery during the day (08:00–15:59)	115	28.2	59	19.5	0.008
Delivery during the evening (16:00–23:59)	141	34.6	152	50.2	< 0.001
Neonatal outcomes					
Pre-term (< 37 weeks)	10	2.5	10	3.3	0.50
Female h	206	50.5	157	51.8	0.73
Macrosomia (≥ 4500 g)	12	2.9	11	3.6	0.60
Apgar 5min < 7	21^f^	5.2	10^g^	3.3	0.22
Umbilical artery pH ≤ 7.05	5^h^	1.2	3	1.0	0.76
Umbilical artery BE ≤ -12.0	6^h^	1.5	3	1.0	0.57
Neonatal intensive care unit admission	65	15.9	39	12.9	0.25
Infection	9	2.2	1	0.3	0.04

a missing values n = 1

b infection n = 2, preeclampsia n = 3, hand presentation n = 1, foot presentation n = 1

c umbilical cord prolapse n = 2, infection n = 1

d endometritis n = 5, cesarean section wound infection n = 3, episiotomy wound infection n = 4, sepsis (Group C Streptococcus) n = 1

e endometritis n = 3, cesarean section wound infection n = 1

f missing values n = 6

g missing values n = 2

h missing values n = 2

**Table 3:** Childbirth experience questionnaire (CEQ) domain scores (N = 711).

	**Nulliparous n = 408**				**Parous n = 303**				** **
	**mean**	**median**	**SD**	**IQR**	**mean**	**median**	**SD**	**IQR**	**p-value**
Own Capacity	2.4	2.4	0.6	0.8	2.7	2.8	0.6	0.9	< 0.001
Professional Support	3.7	4.0	0.5	0.4	3.7	4.0	0.5	0.4	0.18
Perceived Safety	2.9	3.0	0.7	1.0	3.2	3.3	0.7	0.8	< 0.001
Participation	3.2	3.3	0.8	1.3	3.4	3.7	0.7	1.0	< 0.001
Total CEQ	2.9	3.0	0.5	0.7	3.2	3.3	0.5	0.6	< 0.001
Numbers of items responded to	21.8	22.0	1.3	0	21.7	22.0	1.5	0	0.70

Both mean and median numbers shown since some variables are distributed normally and some are not

SD = Standard deviation

IQR = Interquartile range

**Table 4:** Childbirth experience questionnaire (CEQ) item descriptions (N = 711).

		Nulliparous n = 408							Parous n = 303							
	Item number	Total syample per item n = 408	Floor %* (most negative)	Ceiling %* (most positive)	Mean	Median	SD	IQR	Total sample per item n = 303	Floor %* (most negative)	Ceiling %* (most positive)	Mean	Median	SD	IQR	p-value
**Own Capacity**	** **															
Labor and birth went as I had expected	1	405	23.5	14.6	2.4	2.0	1.0	1	301	9.0	24.9	2.9	3.0	0.9	1	< 0.001
I felt strong during labor and birth	2	406	15.0	16.5	2.6	3.0	0.9	1	301	5.6	25.9	3.0	3.0	0.8	2	< 0.001
I felt capable during labor and birth	4	407	11.3	12.5	2.6	3.0	0.8	1	300	5.3	25.7	3.0	3.0	0.8	1	< 0.001
I was tired during labor and birth	5	406	46.3	6.2	1.8	2.0	0.9	1	300	22.3	15.0	2.3	2.0	1.0	1	< 0.001
I felt happy during labor and birth	6	408	12.5	10.0	2.5	3.0	0.8	1	300	10.7	15.7	2.8	3.0	0.8	1	< 0.001
I felt that I handled the situation well	19	403	1.7	43.9	3.3	3.0	0.8	1	300	2.7	54.0	3.4	4.0	0.7	1	0.004
As a whole, how painful did you feel your childbirth was? (visual analog scale, VAS)	20	403	30.5	8.2	2.0	2.0	0.9	1	299	27.4	12.7	2.2	2.0	1.0	2	0.013
As a whole, how much control did you feel you had during childbirth? (VAS)	21	402	51.5	4.7	1.8	1.0	0.9	1	297	32.7	12.8	2.2	2.0	1.0	2	< 0.001
**Professional Support**	** **															
My midwife devoted enough time to me	13	405	1.0	79.5	3.8	4.0	0.5	0	301	0.7	80.1	3.7	4.0	0.6	0	0.96
My midwife devoted enough time to my partner	14	400	1.5	73.0	3.7	4.0	0.6	1	295	1.0	76.9	3.7	4.0	0.6	0	0.22
My midwife kept me informed about what was happening during labor and birth	15	404	1.2	71.3	3.7	4.0	0.6	1	302	0.3	76.5	3.7	4.0	0.6	0	0.12
My midwife understood my needs	16	406	1.2	71.2	3.7	4.0	0.6	1	301	1.0	74.1	3.7	4.0	0.6	1	0.50
I felt very well cared for by my midwife	17	408	0.5	83.5	3.8	4.0	0.5	0	299	1.0	82.6	3.8	4.0	0.5	0	0.73
**Perceived Safety**	** **															
I felt scared during labor and birth	3	408	17.6	21.8	2.5	2.0	1.0	1	300	14.7	27.3	2.7	3.0	1.0	2	0.04
I have many positive memories from childbirth	7	407	11.1	20.9	2.8	3.0	0.9	1	301	4.7	39.5	3.2	3.0	0.8	1	< 0.001
I have many negative memories from childbirth	8	408	16.2	16.4	2.5	3.0	1.0	1	301	6.6	33.6	3.0	3.0	0.9	2	< 0.001
Some of my memories from childbirth make me feel depressed	9	408	14.5	41.7	2.9	3.0	1.1	2	300	6.3	60.3	3.3	4.0	1.0	1	< 0.001
My impression of the team’s medical skills made me feel secure	18	407	2.5	74.7	3.7	4.0	0.7	1	300	1.0	74.7	3.7	4.0	0.6	1	0.93
As a whole, how secure did you feel during childbirth? (VAS)	22	102	8.5	52.5	3.2	4.0	1.0	1	297	9.0	61.5	3.4	4.0	0.9	1	0.02
**Participation**	** **															
I felt I could have a say whether I could be up and about or lie down	10	406	7.1	54.2	3.3	4.0	0.9	1	298	5.4	64.4	3.5	4.0	0.9	1	0.01
I felt I could have a say in deciding my birthing position	11	397	17.4	40.6	2.9	3.0	1.1	2	293	7.8	58.0	3.3	4.0	0.9	1	< 0.001
I felt I could have a say in the choice of pain relief	12	407	2.9	59.5	3.5	4.0	0.8	1	300	4.7	34.3	3.5	4.0	0.8	1	0.15

Higher CEQ scores represent more positive experiences

Both mean and median numbers shown since some variables are distributed normally and some are not

*Percentages higher than 15 indicate unsuitability within the population studied, because if too many respondents choose the extreme response, no distinction between the respondents is possible [18].

SD = Standard deviation

IQR = Interquartile range

**Table 5:** Factors associated with a negative childbirth experience (n = 52; 7.3%).

	Univariable			Multivariable		
	OR	CI (95%)	p-value	OR	CI (95%)	p-value
Nulliparity	1.7	0.9–3.1	0.08	1.2	0.6–2.4	0.63
Need for cervical ripening	1.1	0.5–2.7	0.78	1.0	0.4–2.7	0.97
Oxytocin use in labor induction or augmentation	1.6	0.7–3.6	0.27	1.5	0.6–3.8	0.38
Epidural use	0.8	0.4–1.5	0.44	0.6	0.3–1.2	0.15
Cesarean section	3.3	1.9–5.9	< 0.001	6.7	1.9–9.3	< 0.001
Hemorrhage ≥ 1500 ml in vaginal delivery	1.8	0.7–4.3	0.22	2.8	1.1–7.5	0.03
Induction to delivery interval ≥ 24 h	1.5	0.9–2.7	0.15	0.9	0.4–1.8	0.68
Duration of labor ≥ 12 h	1.5	0.8–2.7	0.21	1.2	0.6–2.4	0.66
Daytime delivery	1.7	0.9–3.1	0.08	1.5	0.9–2.8	0.20

All the variables significantly related to a negative experience in Table 6 were chosen for this multivariable model. All variables used in this final model are shown in the table.

**Table 6:** Factors associated with a negative childbirth experience according to domain.

	Own Capacity (n = 212; 29.9%)						Professional Support (n = 206; 29.1%)						Perceived Safety (n = 111; 15.6%)						Participation (n = 354; 50.2%)					
	Univariable			Multivariable			Univariable			Multivariable			Univariable			Multivariable			Univariable			Multivariable		
	OR	CI (95%)	p-value	OR	CI (95%)	p-value	OR	CI (95%)	p-value	OR	CI (95%)	p-value	OR	CI (95%)	p-value	OR	CI (95%)	p-value	OR	CI (95%)	p-value	OR	CI (95%)	p-value
Nulliparity	2.6	1.8–3.7	< 0.001	1.6	1.0–2.4	0.03	1.2	0.8–1.6	0.34	1.0	0.7–1.5	0.89	2.0	1.3–3.1	0.002	1.1	0.7–1.9	0.72	1.6	1.2–2.2	0.002	1.4	1.0–2.0	0.09
Maternal reason for induction of labor	0.4	0.2–0.8	0.02	0.5	0.2–1.3	0.15	0.5	0.3–1.1	0.09	0.6	0.3–1.3	0.17	0.1	0.01–0.8	0.03	0.1	0.02–1.0	0.05	1.1	0.6–1.9	0.79	1.5	0.8–2.9	0.20
Need for cervical ripening	1.7	1.0–2.9	0.05	1.4	0.8–2.7	0.24	1.6	1.0–2.8	0.07	2.0	1.1–3.5	0.02	2.1	1.0–4.5	0.05	1.7	0.7–3.8	0.24	1.4	0.9–2.3	0.11	1.4	0.9–2.4	0.15
Oxytocin use in labor induction or augmentation	1.6	1.1–2.6	0.02	0.9	0.5–1.5	0.67	1.0	0.7–1.5	0.91	1.2	0.7–1.9	0.56	1.8	1.0–3.2	0.06	1.3	0.7–2.7	0.41	1.8	1.2–2.7	0.002	2.1	1.4–3.5	< 0.001
Epidural or spinal analgesia	2.3	1.4–3.7	< 0.001	1.9	1.1–3.2	0.03	0.6	0.4–0.9	0.01	0.5	0.3–0.8	0.004	1.5	0.9–2.7	0.16	1.2	0.6–2.4	0.53	0.7	0.5–1.0	0.04	0.4	0.3–0.6	< 0.001
Operative vaginal delivery by vacuum extraction	1.2	0.7–1.9	0.46	1.0	0.6–1.8	0.94	1.0	0.6–1.7	0.86	1.3	0.8–2.4	0.31	0.8	0.4–1.5	0.41	0.8	0.4–1.7	0.56	1.3	0.8–2.0	0.32	1.6	1.0–2.7	0.07
Cesarean section	3.7	2.6–5.4	< 0.001	3.2	2.0–5.0	< 0.001	1.9	1.3–2.8	< 0.001	2.2	1.4–3.5	< 0.001	4.6	3.0–7.0	< 0.001	3.7	2.2–6.4	< 0.001	2.5	1.7–3.7	< 0.001	2.6	1.6–4.0	< 0.001
Maternal complication in labor	1.7	1.0–2.9	0.04	1.1	0.6–1.9	0.73	1.3	0.8–2.2	0.26	1.1	0.7–2.0	0.63	2.2	1.2–3.8	0.006	1.4	0.8–2.7	0.25	1.1	0.7–1.7	0.84	0.8	0.5–1.4	0.54
Hemorrhage ≥ 1500 ml in vaginal delivery	1.6	0.9–2.8	0.13	2.2	1.2–4.3	0.02	1.1	0.6–2.1	0.71	1.4	0.7–2.6	0.37	1.5	0.8–3.1	0.23	2.7	1.2–5.9	0.01	1.0	0.5–1.7	0.86	1.1	0.6–2.1	0.68
Induction to delivery interval ≥ 24 h	2.1	1.5–2.9	< 0.001	0.9	0.6–1.4	0.57	1.1	0.8–1.5	0.72	0.6	0.4–1.0	0.05	2.2	1.4–3.3	< 0.001	1.1	0.6–1.9	0.86	1.3	1.0–1.7	0.09	0.8	0.6–1.2	0.33
Duration of labor ≥ 12 h	2.4	1.7–3.5	< 0.001	1.5	1.0–2.3	0.06	1.3	0.9–1.8	0.18	1.2	0.8–1.9	0.33	1.7	1.1–2.6	0.02	1.1	0.5–1.8	0.90	1.1	0.9–1.6	0.49	0.9	0.6–1.4	0.61
Neonate transferred to intensive care unit	1.7	1.1–2.6	0.02	1.3	0.8–2.1	0.32	1.1	0.7–1.8	0.58	0.9	0.5–1.4	0.57	2.3	1.4–3.8	< 0.001	1.7	1.0–2.9	0.06	1.7	1.1–2.6	0.02	1.4	0.9–2.3	0.14
Daytime delivery	2.0	1.4–2.9	< 0.001	1.7	1.2–2.5	0.008	1.9	1.3–2.7	< 0.001	1.8	1.3–2.7	0.002	1.8	1.2–2.8	0.009	1.6	1.0–2.5	0.07	1.2	0.9–1.7	0.25	1.1	0.7–1.6	0.67

Variables in this final multivariable model are chosen by assessing the previous literature and by using stepwise logistic regression analysis. All variables used in this final model are shown in the table.

## Results

A total of 1080 women were recruited, of whom 750 returned the survey questionnaire (response rate 69.4%, comprising 71.4% nulliparous women and 67.1% parous women, p = 0.14), and 711 met the study criteria. In the final study population of 711 women, 408 (57.4%) were nulliparous and 303 (42.6%) were parous (Fig 1).

The characteristics of the study population are shown in Table 1. The mean maternal age was 33.6 (SD 5.0) years, the median maternal pre-pregnancy body mass index was 24.0 (IQR 6.2) kg/m^2^, and the mean gestational age at labor induction was 40.1 (SD 1.5) weeks. The nulliparous women more often had a late term pregnancy (≥ 41 weeks) and a Bishop score < 3 at the start of labor induction, while the parous women more often had gestational diabetes (Table 1). The nulliparous women underwent cervical ripening more often than the parous women (Table 1). The most commonly used method for cervical ripening was a balloon catheter (in 244 [59.8%)] nulliparous women and in 218 (71.9%] parous women, p < 0.001]. The nulliparous women more often had labor induction for post-term pregnancy and pre-labor rupture of membranes, while the parous women more often had labor induction for diabetes or maternal or fetal reasons (Table 1).

The delivery and neonatal outcomes are shown in Table 2. Oxytocin use in labor induction or augmentation and epidural or spinal analgesia were more common in the nulliparous women than the parous women (Table 2). The cesarean section rate was 22.8% (n = 162) and was higher in the nulliparous than in the parous women (Table 2). The indications for cesarean delivery did not differ between the groups (Table 2). The rates of vaginal vacuum extraction delivery, episiotomy, and intrapartum infection were higher among the nulliparous women than the parous women (Table 2). The nulliparous women most often delivered during the night, and the parous women most often delivered during the evening (Table 2). The median induction to delivery interval (31.2 [IQR 25.0] hours vs. 14.1 [IQR 18.7] hours, p < 0.001) and the median duration of labor (10.8 [IQR 7.5] hours vs 4.7 [IQR 4.1] hours, p < 0.001) were longer the in nulliparous women compared with the parous women. The mean birth weight was 3591 (SD 505) grams, and no differences in neonatal outcomes between the nulliparous and the parous women were found (Table 2).

The mean and median scores of the four CEQ domains, the total CEQ score, and the number of items responded to are presented in Table 3 according to parity. The mean total CEQ score was 3.0 (SD 0.5), lower in the nulliparous than in the parous women (Table 3). Of the CEQ domain scores, “Own Capacity,” “Perceived Safety,” and “Participation,” were all lower in the nulliparous women compared with the parous women (Table 3). The domain score for “Professional Support” did not differ between the groups, nor did the mean number of items responded to (Table 3).

Table 4 shows the individual items of the CEQ with floor and ceiling effects. The nulliparous women had lower scores in all CEQ items in “Own Capacity” and in all but one item in “Perceived Safety” and “Participation”. No differences in individual items in “Professional Support” were detectable.

We found ceiling effects in three of the four CEQ domains (“Professional Support,” “Perceived Safety,” and “Participation”): all items within these domains had > 15% of scores in the most positive alternatives in both the nulliparous and the parous women (Table 4). The domain “Own Capacity” showed a ceiling effect in 25.0% (two of eight) of the items in the nulliparous and 62.5% (five of eight) of the items in the parous women (Table 4). The CEQ was better in distinguishing negative responses, as “Professional Support” showed no floor effect, and in “Perceived Safety” and “Participation,” floor effects were only seen in the nulliparous women with 33.3% (two of six and one of three, respectively) of the items (Table 4). In “Own Capacity”, a floor effect was detectable in 50.0% (four of eight) of the items in the nulliparous and 37.5% (three of eight) of the items in the parous women (Table 4).

**Fig 2:**
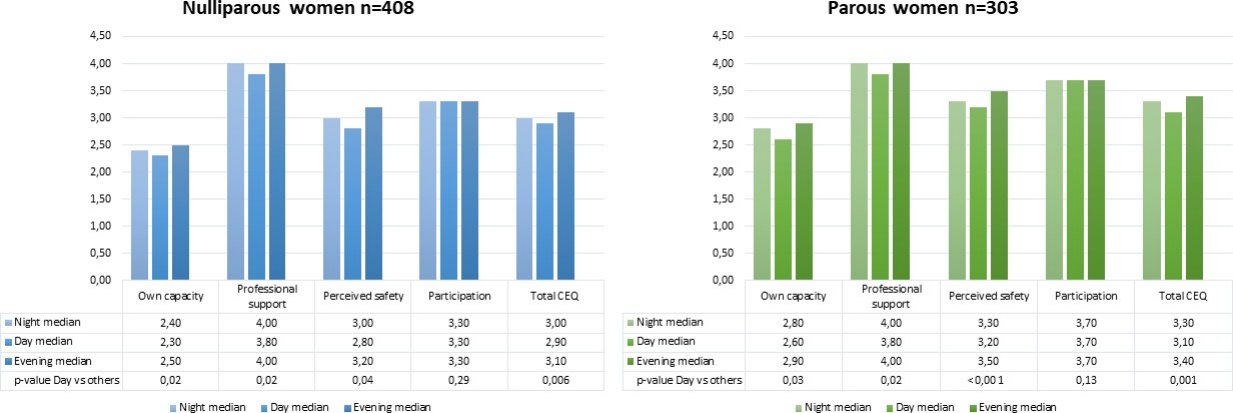
Effect of time of day in childbirth experience questionnaire (CEQ) N = 711.

The effect of time of day on the CEQ scores is presented in Fig 2 according to parity. Lower scores in daytime delivery are observable for both the nulliparous and the parous women compared with other times of day in “Own Capacity,” “Professional Support,” “Perceived Safety,” and the total CEQ score.

A negative childbirth experience, as defined by the most negative quartile of scores given in all domains of the CEQ, was detected in 7.3% (52 of 711) of the women (8.8% [36 of 408) nulliparous and 5.3% [16 of 303] parous, p = 0.08). In the multivariable analysis, a negative experience was associated with a cesarean section and a hemorrhage ≥ 1500 ml in a vaginal delivery (Table 5).

Table 6 shows the multivariable analyses of the CEQ domain scores separately. A cesarean section was associated with a negative experience in all domains, and a hemorrhage ≥ 1500 ml in a vaginal delivery was associated with a negative experience in “Own Capacity” and “Perceived Safety” (Table 6). Nulliparity negatively affected “Own Capacity,” and daytime delivery negatively affected “Own Capacity” and “Professional Support” (Table 6). Cervical ripening was associated with a negative experience in “Professional Support,” and oxytocin use in labor induction or augmentation was associated with a negative experience in “Participation” (Table 6). Use of epidural or spinal analgesia affected negatively “Own Capacity” but protected against a negative experience in “Professional Support” and “Participation” (Table 6).

## Discussion

Overall, women whose labor was induced experienced their labor and birth as a relatively positive experience, with a mean CEQ score of 2.9 for the nulliparous women and 3.2 for the parous women (scale 1–4). However, 7.3% (52 of 711) of the women had a negative childbirth experience.

In the multivariable analyses, nulliparity was associated with a negative experience only in the domain “Own Capacity.” As parous women more often reported feeling strong and capable and that labor and birth went as expected, supporting nulliparous women in these aspects could be beneficial. Hospital staff were given very favorable scores in the study; thus, more emphasis should perhaps be placed on antenatal counseling. Prenatal classes offered to nulliparous women in our maternity care units are brief and medically oriented, whereas many private paid classes focus on the woman´s inner strength, nonmedical ways of pain relief, relaxation, and making labor an empowering experience. In earlier studies, birth preparedness has been associated with higher satisfaction, improvement of women´s sense of control and confidence in giving birth [23, 24]. Thus, we suggest offering more thorough prenatal classes to all nulliparous women.

A cesarean section was the factor most associated with a negative childbirth experience, in line with earlier studies [11, 15]. Since a cesarean section leads to an increase in risks for both the mother and her neonate in the present and subsequent pregnancies and labor while also being more costly compared with vaginal delivery [25, 26], safely preventing a primary cesarean is considered essential in modern obstetric practice [21]. When these factors are considered alongside the results of studies on the childbirth experience, preventing unnecessary cesarean sections appears even more important. Interestingly, operative vaginal delivery was not associated with a negative childbirth experience in this study. Perhaps in this setting of labor induction, the additional need for the assistance of an obstetrician was noted more mildly than in a labor of spontaneous onset [7].

Of all the maternal complications investigated in our study, only a postpartum hemorrhage ≥ 1500 ml in a vaginal delivery was associated with a negative childbirth experience. Our results agree with previous research in which postpartum hemorrhage has been associated with dissatisfaction with childbirth [7, 12]. In our study, a postpartum hemorrhage was associated with a negative experience in the domains “Own Capacity” and “Perceived Safety.” This may be due to the physiological effects related to hemorrhage (tachycardia, tachypnea, sweating, and weakness) and the hurried efforts of staff to control the hemorrhage, both of which can make the woman feel out of control and unsafe. Effective prevention of postpartum hemorrhage and empathetic support after an acute emergency are thus important.

Daytime delivery negatively affected “Own Capacity” and “Professional Support,” which was surprising since daytime delivery has previously been noted to be favorable [16, 17]. Possibly, other daily activities in the labor and delivery unit could affect this phenomenon, preventing staff from attending to the woman in labor as preferred. Furthermore, midwives change shifts in the afternoon; thus, the treating midwife may change during labor. We also hypothesize that women delivering during daytime have possibly spent the previous night awake with painful contractions due to induction. Quality care is only possible with adequate staffing [27]; and since midwives and obstetricians are the key professionals in improving the childbirth experience [1, 28, 29], hospital processes and staffing should be organized accordingly.

Cervical ripening was associated with a negative experience in “Professional Support.” At our hospital, most women with a balloon catheter are offered outpatient protocol, and as most women undergoing cervical ripening had a balloon catheter, it is likely that most were treated as outpatients. Although the opportunity to stay home during early induction is often positively noted [30, 31], it could also be experienced as being left without appropriate support.

The use of epidural or spinal analgesia negatively affected “Own Capacity,” corresponding to an earlier Swedish study [32], but it was a protective factor against a negative experience in “Professional Support” and “Participation.” In the abovementioned study, this protective effect was not seen, but unlike in our study, they observed a negative effect in “Perceived Safety.” At our hospital, when women are asked about their wishes for labor and delivery, many prefer nonmedical pain relief methods. As labor progresses, most opt for epidural or spinal analgesia. On the one hand, this change of preference may negatively affect the perception of self-capability, but on the other hand, as the woman is not concentrating solely on enduring labor pain, other efforts by the staff may be more easily noticed and making informed choices on labor management options becomes possible.

In previous studies of women with mainly spontaneous labor onset, oxytocin use has been shown to affect childbirth experience negatively [11, 14]. If CEQ domains are analyzed separately, “Own Capacity” is negatively affected [8, 13, 14, 32], and in “Perceived Safety,” augmentation of labor has shown mixed, but mainly negative, results [8, 13, 14, 32]. In our study, oxytocin use was associated with a negative experience only in “Participation.” This negative association with “Participation” was also found in the original validation study of CEQ, but not in other studies [8, 13, 14, 32]. In our opinion, these differences in results may imply that induced vs. spontaneous onset of labor modifies women’s experience of some aspects of labor itself. Our hypothesis for the results showing negative experiences in “Participation” is that intravenous lines prevent free movement during labor and make it an easier option to stay in the bed. As oxytocin use is rarely avoidable in nulliparous women undergoing labor induction, helping women to be active despite intravenous lines could improve their perception of being active participants in the labor process.

The strengths of our study are a relatively large sample size, detailed medical records, and a uniform protocol of labor induction and labor treatment at our hospital. The weaknesses include a possible selection bias, since not all the women undergoing labor induction participated, and 30.6% of the recruited women did not return the questionnaire. Also, not including women with spontaneous onset of labor or elective cesarean delivery could be seen as a weakness. Furthermore, we did not have data on outpatient cervical ripening or the presence and identity of a companion. However, in this study, 685 (97.7%) women answered the item “My midwife devoted enough time to my partner,” implying that these women had someone with them for support.

The CEQ showed a ceiling effect, reflecting difficulty in distinguishing a positive childbirth experience using this questionnaire. In this setting, the CEQ was better at distinguishing a negative childbirth experience. The CEQ2 [33], an updated version of the original CEQ used in this study, differs somewhat in the domains “Professional Support” and “Participation,” and it has been used in a population with a very high rate of labor induction (64.0%) [34]. However, at the time of this study, it was not available in Finnish. In future studies on labor induction, using the updated CEQ2 would perhaps be beneficial.

## Conclusions

Nulliparous women undergoing labor induction are at risk of a negative childbirth experience, mainly due to labor and delivery-related factors, similar to parous women. However, nulliparous women´s perception of their own capability and preparedness for labor and delivery may perhaps be enhanced by more thorough childbirth coaching and support from hospital staff during the whole process of labor induction, labor, delivery, and the postpartum period. An effective labor induction protocol with as high a rate of vaginal delivery as possible and preparedness to promptly respond to postpartum hemorrhage are the key components of medical interventions in avoiding a negative childbirth experience. However, aiming for daytime delivery is not considered a priority.
